# Increasing Antimicrobial Resistance in World Health Organization Eastern Mediterranean Region, 2017–2019

**DOI:** 10.3201/eid2804.211975

**Published:** 2022-04

**Authors:** Maha Talaat, Bassem Zayed, Sara Tolba, Enjy Abdou, Mohamed Gomaa, Dana Itani, Yvan Hutin, Rana Hajjeh

**Affiliations:** World Health Organization Eastern Mediterranean Region, Cairo, Egypt

**Keywords:** antimicrobial resistance, AMR, bloodstream infections, infection prevention and control, antimicrobial stewardship, bacteria, World Health Organization, Eastern Mediterranean Region, antimicrobial drug resistance

## Abstract

To better guide the regional response to antimicrobial resistance (AMR), we report the burden of AMR over time in countries in the World Health Organization Eastern Mediterranean Region. To assess the capacities of national infection prevention and control and antimicrobial stewardship programs, we analyzed data on bloodstream infections reported to the Global Antimicrobial Resistance Surveillance System during 2017–2019, data from 7 countries on nationally representative surveys of antimicrobial prescriptions, and data from 2 regional surveys. The median proportion of bloodstream infections was highest for carbapenem-resistant *Acinetobacter* spp. (70.3%) and lowest for carbapenem-resistant *Escherichia coli* (4.6%). Results of the regional assessments indicate that few countries have capacities for infection prevention and control and antimicrobial stewardship programs to prevent emergence and spread of AMR. Overall, the magnitude of the problem and the limited capacity to respond emphasize the need for regional political leadership in addressing AMR.

Antimicrobial resistance (AMR) is a global crisis and one of the world’s most complex challenges, threatening a century of health progress. AMR affects human and animal health and poses a serious threat to reaching sustainable development goals and food security. Drug-resistant infections account for 700,000 deaths globally each year and could cumulate to 10 million by 2050 if no sustained efforts to contain AMR are implemented (*1*–*3*).

The Eastern Mediterranean Region (EMR) of the World Health Organization (WHO) consists of 21 countries and the occupied Palestinian territory (731 million persons in 2021) (*4*). The region is diverse; and social, economic, and demographic conditions are challenging. Nearly two thirds of the countries are affected by conflicts, wars, and population displacement, posing grave implications for health and severe disruption of health systems (*5*). Factors contributing to the emergence and spread of AMR in the EMR include the high burden of infectious diseases; weak health and surveillance systems; inadequate regulatory frameworks; poor infection prevention and control (IPC) in healthcare facilities; limited capacities of microbiology laboratories; lack of access to quality-assured antimicrobial drugs for humans and animals; poverty; inadequate access to water, sanitation, and hygiene; and limited antimicrobial stewardship (AMS) programs (*6*). Antimicrobial drugs are available over the counter, and self-medication is a common practice in most countries. Inappropriate prescription practices among physicians are widespread. Antimicrobial drugs are used to compensate for the lack of basic public health infrastructure (e.g., vaccination coverage and IPC) (*7*,*8*).

WHO identified surveillance as 1 of the 5 strategic priorities of the global and national AMR action plans (*9*,*10*). Because most countries did not have good quality AMR data, in 2015, WHO launched the Global Antimicrobial Resistance Surveillance System (GLASS, https://www.who.int/glass). To measure the regional AMR burden and generate quality data, WHO supported countries to establish and enhance national AMR surveillance. We evaluated the burden of AMR for selected serious resistant bacterial infections reported to WHO through GLASS over 3 years (2017–2019), along with the regional capacities of AMS and IPC programs. We also explored the challenges faced by countries responding to AMR and propose priority actions to advance the AMR control agenda in the EMR.

## Methods

### GLASS Design

Each country assigned a variable number of sentinel surveillance sites on the basis of good quality microbiology laboratories. The AMR data are generated through processing of specimens (e.g., blood, urine, stool) collected for clinical purposes. Isolating and identifying bacteria through antimicrobial sensitivity testing (AST) formed the basis of reporting; countries used either Clinical Laboratory Standards Institute (https://clsi.org) or European Committee on Antimicrobial Sensitivity Testing (https://www.eucast.org) guidelines. Surveillance sites used WHONET software (*11*), which was adapted to improve data entry and reporting to GLASS through an aggregated format. Some countries reported demographic and epidemiologic variables such as age, sex, and origin of infection. However, these data were incomplete (*10*), and we did not use them for analysis.

### AMR Data Collection, Case Definitions, and Data Analysis

For this article, we report only bloodstream infections (BSIs) caused by resistant organisms and used the data reported to GLASS by 11–14 countries during 2017–2019 (Appendix). We prioritized BSIs because they are among the most serious and life-threatening infectious conditions and are used as the main sustainable development goal indicator for AMR. The presence of a pathogen in blood samples is used as a proxy for BSI in a patient. We report BSIs caused by carbapenem-resistant *Acinetobacter* spp. (CRAsp)*,* third-generation cephalosporin (3GC)–resistant *Enterobacteriaceae* (*Escherichia coli* and *Klebsiella pneumoniae*), methicillin-resistant *Staphylococcus aureus* (MRSA), and carbapenem-resistant *Enterobacteriaceae* (CRE) (*E. coli* [CREC] and *K. pneumoniae* [CRKP]).

To calculate the proportion of patients with BSIs caused by resistant pathogens, we used as a numerator the number of patients with BSIs caused by one of the specific resistant pathogens and as the denominator the total number of patients with BSIs that were tested by AST (susceptible, intermediate, resistant) for the same pathogen. For example, we calculated the proportion of patients with BSIs caused by CRAsp by dividing the number of patients with BSIs caused by CRAsp by the number of patients with BSIs caused by *Acinetobacter* spp. with AST results (susceptible, intermediate, or resistant) for carbapenems. We used box-and-whisker plots to display the proportion of BSIs caused by specific resistant pathogens. We also described the distribution of resistance over time with a line graph and the geographic distribution with maps.

### Antimicrobial Drug Prescriptions among Hospitalized Patients

Seven countries participated in a standardized regional point prevalence survey that measured antimicrobial drug use among hospitalized patients. Countries selected a nationally representative sample of healthcare facilities and assigned national teams to collect the data. WHO trained the data collection teams to ensure standardization and collection of good quality data. We calculated the prevalence of antimicrobial drug use by dividing the number of patients prescribed >1 antimicrobial drug at the time of the survey over the total number of inpatients surveyed.

### IPC Program Capacity Assessment

To conduct a regional survey at the end of 2019 and assess the national IPC programs in countries, we used the WHO IPCAT2 tool (*12*). The main purpose of the IPCAT2 tool is to describe the status of the national IPC activities according to WHO guidelines (*13*) and identify strengths and weaknesses to plan for improvement. We collected data through personal interviews with representatives at national IPC focal points or their alternatives.

### AMS Programs Capacity Assessment

To assess the preparedness of countries regarding their national AMS programs, we completed a regional survey in early 2020 by using the WHO AMS assessment tool (*14*). The tool assesses 4 core elements of AMS programs: 1) national plans and strategies; 2) regulations and guidelines; 3) awareness, training, and education; and 4) supporting technologies and data. We collected the data through virtual personal interviews with national AMR/AMS representatives in each country.

## Results

### AMR Surveillance

#### Reporting to GLASS

The number of countries and health facilities reporting to GLASS increased over time. For 2017, a total of 235 health facilities in 12 countries reported data on any type of infection, increasing to 373 health facilities in 15 countries in 2018 and to 527 health facilities in 18 countries in 2019. The number of countries that reported BSIs to GLASS also increased; 11 countries reported BSIs in 2017, 12 in 2018, and 14 in 2019. Also, the number of reported BSIs caused by priority pathogens increased from 6,957 BSIs in 2017 to 16,454 in 2018 and 23,104 in 2019 (*15*,*16*).

#### AMR Data

**Figure 1:**
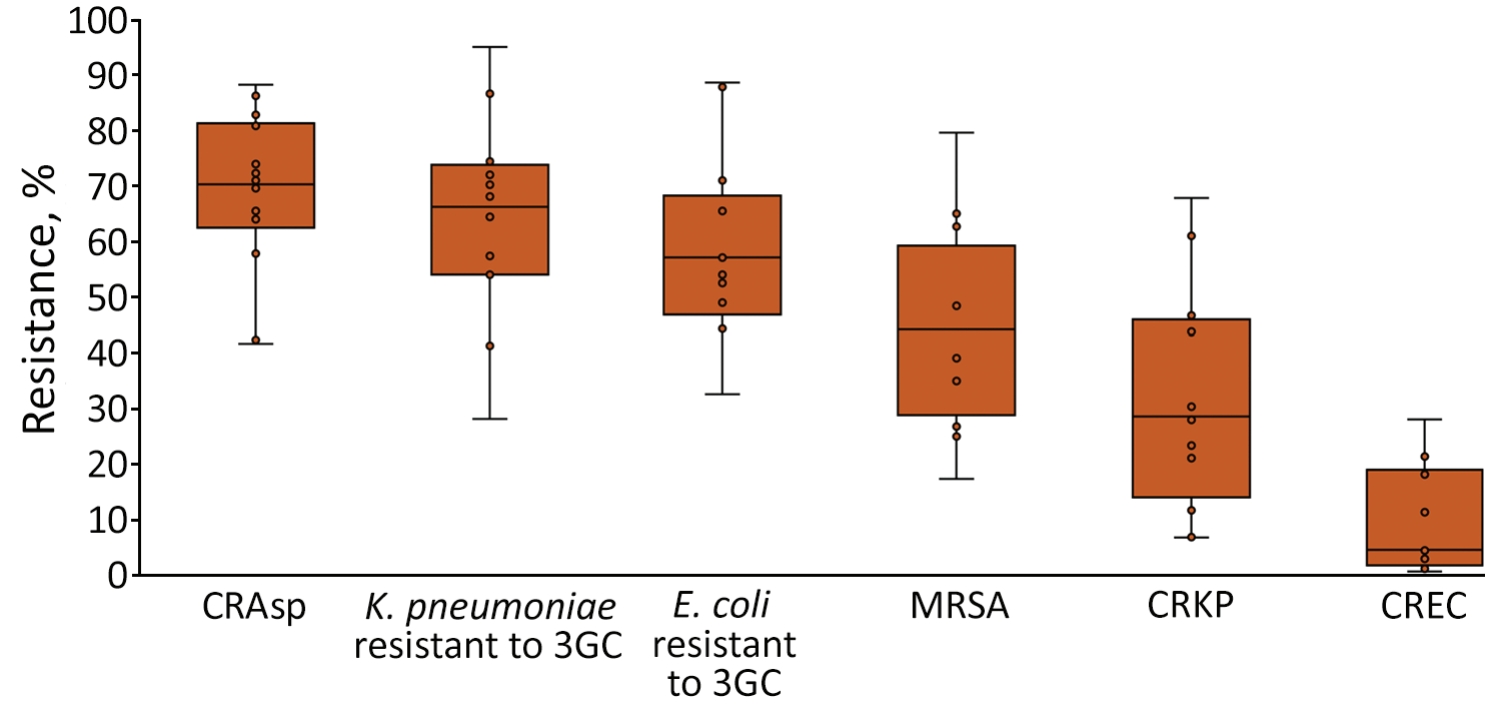
Proportion of patients with bloodstream infections caused by antimicrobial resistant pathogens in 14 World Health Organization Eastern Mediterranean Region countries. Data from the Global Antimicrobial Resistance Surveillance System (https://www.who.int/glass) for 2019. Each dot represents the percentage of patients with resistant organisms in a country. Horizontal lines within boxes indicate medians, box tops and bottoms indicate interquartile ranges (middle 50% of data), and error bars (upper and lower whiskers) represent scores outside the middle 50%. CRAsp, carbapenem-resistant *Acinetobacter* spp., CREC, carbapenem-resistant *Escherichia coli*; CRKP, carbapenem-resistant *K. pneumoniae*; *E. coli*, *Escherichia coli*; *K. pneumoniae*, *Klebsiella pneumoniae*; MRSA, methicillin-resistant *Staphylococcus aureus*; 3CG, third-generation cephalosporins.

**Figure 2:**
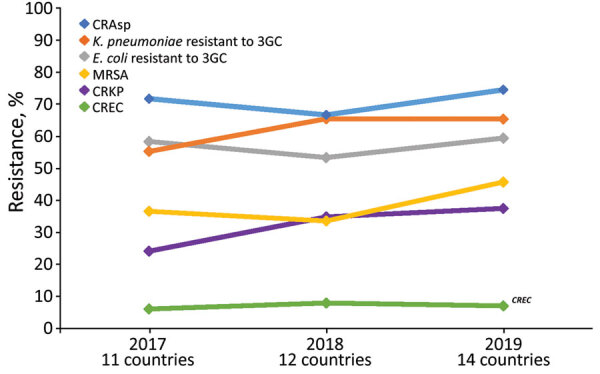
Proportion of patients with bloodstream infections caused by antimicrobial resistant pathogens in 11–14 World Health Organization Eastern Mediterranean Region countries. Data from the Global Antimicrobial Resistance Surveillance System (https://www.who.int/glass) for 2017–2019. CRAsp, carbapenem-resistant *Acinetobacter* spp.; CREC, carbapenem-resistant *Escherichia coli*; CRKP, carbapenem-resistant *K. pneumoniae*; *E. coli*, *Escherichia coli*; *K. pneumoniae*, *Klebsiella pneumoniae*; MRSA, methicillin-resistant *Staphylococcus aureus*; 3CG, third-generation cephalosporins.

**Figure 3:**
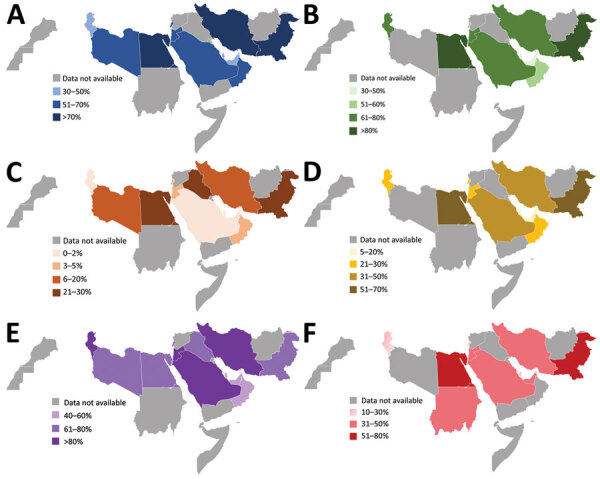
Proportion of patients with bloodstream infections caused by antimicrobial resistant pathogens in World Health Organization Eastern Mediterranean Region countries. Data from the Global Antimicrobial Resistance Surveillance System (https://www.who.int/glass) for 2019. A) Caused by 3GC-resistant *Escherichia coli,* 14 countries. B) Caused by *K. pneumoniae* resistant to 3GC, 12 countries. C) Caused by carbapenem-resistant *E. coli*, 14 countries. D) Caused by carbapenem-resistant Klebsiella *pneumoniae*, 13 countries. E) Caused by carbapenem-resistant *Acinetobacter* spp., 12 countries. F) Caused by methicillin-resistant *Staphylococcus aureus*, 12 countries.

In 2019, the median proportion of patients with BSIs caused by CRAsp was highest at 70.3% (IQR 62.4%–81.3%), followed by *K. pneumoniae* resistant to 3GC (66.3%, IQR 54.0%–3.8%). The lowest median proportion was for CREC (4.6%, IQR 1.8%–18.2%). The proportion of BSIs caused by resistant pathogens varied widely across countries: 41.7%–88.2% for CRAsp, 28.2%–95.0% for 3GC-resistant *K. pneumoniae*; 32.6%–88.6% for 3GC-resistant *E. coli*; 17.4%–79.6% for MRSA, 6.8%–67.8% for CRKP; and 0.7%–28.1% for CREC (Figure 1). Although assessing trends with only 3 years of data is difficult, especially with the changes in number of reporting countries and surveillance sites, the proportion of resistance tended to increase over time, from 71.4% in 2017 to 74.5% in 2019 for CRAsp, from 55.3% in 2017 to 65.4% in 2019 for 3GC-resistant *K. pneumoniae*, from 36.6% in 2017 to 45.8% in 2019 for MRSA, and from 24.2% in 2017 to 37.5% in 2019 for CRKP. 3GC-resistant *E. coli* increased minimally from 58.4% in 2017 to 59.5% in 2019, and CREC increased from 6.1% in 2017 to 7.1% in 2019 (Figure 2). Egypt and Pakistan had the highest proportion of resistance for 5 of the 6 resistant pathogens; Qatar and United Arab Emirates had the lowest proportion of resistance for 5 (Qatar) and 3 (United Arab Emirates) of the 6 resistant pathogens (Figure 3).

### Antimicrobial Drug Prescriptions among Hospitalized Patients

A total of 128 hospitals in 7 countries (Jordan, Sudan, Pakistan, United Arab Emirates, Tunisia, Lebanon, and Iraq) participated in the prevalence survey for antimicrobial drug use among hospitalized patients. Among the 16,551 patients hospitalized, 8,814 (53.3%) received >1 antimicrobial agent, ranging from 38.7% to 77.7% across countries. The most common indication for prescribing antimicrobial drugs in all countries was treatment of community-acquired infections. Respiratory infections accounted for the largest proportion of infections treated. The top 3 antimicrobial drugs prescribed were 3GC (22.6%), imidazoles (9%), and carbapenems (8.5%). According to the WHO Access, Watch or Reserve (AWaRe) classification of antibiotics (*17*,*18*), only 34% of the prescribed antimicrobial drugs were from the access group, 61% were from the watch group, and 5% were from the reserve group.

### IPC Program Assessment

Among the 22 countries in the region, 13 (59%) had a national IPC program established within their ministries of health. However, for 8 (61.5%) of the 13 countries with an IPC program, the existing structures were not functioning and did not implement IPC policies and procedures in healthcare facilities. Nine (40%) of the 22 countries had developed national IPC guidelines within the past 5 years, 7 had active IPC education programs, and 6 had either multimodal strategies or national IPC monitoring plans (Table 1).

**Table 1:** Key elements of national IPC core components in 22 countries of the World Health Organization Eastern Mediterranean Region, 2019*

National IPC core components	Countries, no. (%)
IPC focal point/group	13 (59)
Evidence-based national IPC guidelines within past 5 y	9 (41)
National IPC education and training program	7 (32)
National healthcare-associated infection surveillance program	5 (23)
National IPC multimodal improvement strategies	6 (27)
National monitoring/auditing of IPC practices and feedback	6 (27)

*IPC, infection prevention and control.

### AMS Assessment

We assessed national AMS core capacities for 20 of the 22 EMR countries. Only 1 (5%) country had dedicated funding for AMS, 4 (20%) had established national AMS technical working groups, and 1 (5%) had developed an AMS implementation plan. With regard to regulations and clinical guidelines, 13 (65%) countries reported having an Essential Medicines List, 2 of which adopted the AWaRe classification; 5 (25%) had treatment guidelines, and 10 (50%) had a prescription-only sale policy for antibiotics, of which only 5 enforced this policy (Table 2).

**Table 2:** Implementation status of select core elements of national AMS programs in 20 countries in the World Health Organization Eastern Mediterranean Region, 2020*

Activities	Countries, no. (%)
National core elements, national plans, and strategies	
Dedicated funding for AMR and AMS activities	1/20 (5)
Establishment of AMS technical working group with defined terms of references	4/20 (20)
Development of AMS implementation plan with short-, medium-, and long-term goals	1/20 (5)
Regulations and guidelines	
Presence of national Essential Medicines List	13/20 (65)
National EML adopts AWaRe classification	2/13 (15.5)
Development of updated treatment guidelines for common infections	5/20 (25)
Existing treatment guidelines integrate the AWaRe classification	1/5 (20)
Presence of prescription only sale policy for antimicrobial drugs	10/20 (50)
Enforcement of prescription only sale policy for antimicrobial drugs	5/10 (50)
Awareness, training, and education	
In-service training for AMS teams on AMS and antimicrobial drug prescribing	0/20
In-service training for healthcare professionals on AMS and antimicrobial drug prescribing	2/20 (10)
AMS criteria for hospitals accreditation, set by ministry of health	4/20 (20)

*AMR, antimicrobial resistance; AMS, antimicrobial stewardship; AWaRe, Access, Watch, or Reserve.

## Discussion

Of the 22 EMR countries, 20 developed their national AMR action plans in alignment with the global AMR action plans. Since 2017, several countries in the region started generating data on AMR and antimicrobial drug use. Hence, detection and surveillance capabilities increased in most countries along with awareness of the scope and complexity of AMR.

The median percentage of patients with BSIs caused by CRAsp was highest at 70.3%. This figure is extremely high compared with the percentage in the United States, where CRAsp among healthcare-associated infections is at 33.9% (*19*); in European Union countries, 32.6% of *Acinetobacter* spp. isolates identified in blood or cerebrospinal fluid were resistant to carbapenems (*20*). CRAsp is a high-threat pathogen; resistant clones are spreading in healthcare settings. Transmission is exacerbated by limited implementation of IPC (*21*). CRAsp and CREs have become resistant to nearly all available antimicrobial drugs, contributing to patient deaths and case-fatality rates >50% (*22*,*23*).

The data for antimicrobial drug prescriptions among hospitalized patients pointed to high use of 3GC and carbapenems, which may explain the high levels of resistance for these drugs (*K. pneumoniae* resistant to 3GC and CREs). The overall prevalence of antimicrobial drug use in the 7 (53.5%) countries that implemented the point-prevalence survey is lower than that in some countries in Africa (Ghana 70.7%, Nigeria 80%) (*24*,*25*), similar to the prevalence for Latin America (49.5%) (*26*) but higher than that for countries in Europe (*27*). The most common indication for antimicrobial drug prescription in the EMR as well as in several countries in Europe and Africa is treatment of community-acquired infections (*25*,*27*). However, in the EMR, 3GC are the most prescribed antimicrobial drugs, and in Europe, penicillins with β-lactamase inhibitors are most commonly prescribed (*27*).

AMS programs prevent further emergence of resistance. In the EMR, these programs are still in their infancy but are evolving with various progress among countries. Only 5 countries in the region are enforcing a prescription-only sale policy for antimicrobial drugs in pharmacies, and 2 countries are adopting AWaRe classification in their national Essential Medicines List to increase the use of the Access group of antimicrobial drugs (first or second empiric choice) for common infections (*17*,*18*). Legislation to enforce this policy must be combined with adequate access to universal health coverage. Despite the high AMR burden, major barriers for AMS implementation exist in the EMR: limited numbers of infectious disease and clinical pharmacy experts in several countries, limitations in microbiological diagnostic capacities, lack of national AMR governance including AMS, knowledge gaps regarding optimum antimicrobial drug use across healthcare providers, insufficiently staffed and overcrowded healthcare systems in some countries, and absence of information technology (including electronic hospital records and clinical decision support systems) to monitor antimicrobial use.

After resistant organisms have emerged, IPC programs are essential for preventing spread. Unfortunately, unlike other preventive and curative interventions, IPC has never been an integrated function of healthcare systems. IPC programs were developed mainly to respond to global or national infectious disease epidemics or pandemics (e.g., bloodborne pathogens, Middle East respiratory syndrome coronavirus, pandemic influenza virus, severe acute respiratory syndrome coronavirus 2). IPC has progressed in several high-income countries, whereas implementation in low- and middle-income countries remains limited and compliance with IPC measures is often low (*28*). Decision makers rarely recognize the role of IPC as a health system–strengthening element with cross-cutting value for AMR response and prevention or control of other infectious diseases. The coronavirus disease pandemic led to recognition of the value of IPC, but many countries in the EMR have yet to establish or enhance their IPC national programs. Although investing in IPC will need resources, the coronavirus disease pandemic demonstrated that such investment will be highly cost-effective for preventing spread of infection among patients and healthcare workers in addition to reducing infections caused by drug-resistant strains (*29*,*30*).

Among limitations of these AMR surveillance data, the increase in number and type of reporting healthcare facilities over time for some countries could affect the proportion of drug-resistant infections reported. First, use of routine clinical data has the potential to overestimate resistance because of the tendency to culture specimens of patients experiencing treatment failure. Second, most AMR data are driven by hospitals with limited understanding of AMR data at the community level. Third, although countries were encouraged to report demographic and clinical characteristics, the completeness of these data is limited for most reporting countries. Fourth, reporting only BSIs does not reflect the complete spectrum of AMR. Last, reporting aggregated data from countries prevents detailed epidemiologic analysis.

In conclusion, the prevalence of AMR in EMR countries is high, and the continued increase threatens health security in the region. AMS programs that prevent emergence of AMR and IPC programs that reduce spread are still developing with variable capacities among countries. This situation calls for political engagement and leadership. EMR countries need to accelerate implementation of the national AMR plans with effective national AMR governance systems, including highly specialized human resources, adequate funding, and empowerment of responsible staff at all levels. Countries within the EMR must continue to enhance and expand their national AMR surveillance programs with a focus on strengthening microbiology laboratories and to fully implement and strengthen AMS and IPC with a focus on safety and quality of services, especially in countries with weak health systems (*1*,*3*,*6*,*14*,*31*). Last, legislation to promote antimicrobial drug use and IPC in this region is urgently needed.

Appendix Supplemental data for study of increasing antimicrobial resistance in the World Health Organization Eastern Mediterranean Region, 2017–2019.
